# ICTV Virus Taxonomy Profile: Bacilladnaviridae 2026

**DOI:** 10.1099/jgv.0.002321

**Published:** 2026-09-29

**Authors:** Arvind Varsani, Yuji Tomaru, Kenta Okamoto, Anna Munke, Mart Krupovic

**Affiliations:** 1 School of Life Sciences, Arizona State University, Tempe, Arizona, USA; 2 Environmental and Fundamental Research Department, Fisheries Technology Institute, Hirosima, Japan; 3 Department of Cell and Molecular Biology, Uppsala University, Uppsala, Sweden; 4 Department of Chemistry, Lund University, Lund, Sweden; 5 Cell Biology and Virology of Archaea Unit, Institut Pasteur, Université Paris Cité, Paris, France

**Keywords:** algal viruses, *Bacilladnaviridae*, *Cressdnaviricota*, *Floreoviria*, ICTV Report, taxonomy

## Abstract

*Bacilladnaviridae* is a family of viruses with single-stranded, circular DNA genomes that range from 3.4 to 6 kb and contain a short double-stranded region of 0.5–1 kb. The genome is replicated by the rolling-circle mechanism. Bacilladnavirids encode a jelly-roll capsid protein which forms non-enveloped icosahedral capsids with a *T*=3 symmetry. All cultured bacilladnavirids infect diatoms, a group of unicellular algae (Stramenopiles), and cause lysis of the host cells. This is a summary of the International Committee on Taxonomy of Viruses (ICTV) Report on the family *Bacilladnaviridae*, which is available at ictv.global/report/bacilladnaviridae.

## Virion

Bacilladnavirids form non-enveloped, icosahedral capsids with *T*=3 symmetry [1, 2] (Table 1; Fig. 1a). The capsid protein (CP) subunits adopt a jelly-roll fold. Within the mature capsid, the outer layer of the genome adopts a partially spooled arrangement (Fig. 1b) [1, 2]. The overall capsid architecture appears to be conserved across the family, although subtle variation in capsid organization may exist [3].

**Table 1.** Characteristics of members of the family *Bacilladnaviridae*

Example	Chaetoceros salsugineum DNA virus 1 (AB193315), species *Protobacilladnavirus chasesal*
Virion	Non-enveloped, icosahedral *T*=3 symmetry, 35 nm in diameter
Genome	Circular, positive-sense DNA of 3.4–6.0 kb, with a partially double-stranded region
Replication	Nuclear, rolling-circle mechanism, lytic
Translation	mRNAs produced via DNA transcription
Host range	Protists (diatoms)
Taxonomy	Realm *Floreoviria*, kingdom *Shotokuvirae*, phylum *Cressdnaviricota*, class *Arfiviricetes*, order *Baphyvirales*: >6 genera, >21 species

**Fig. 1.**
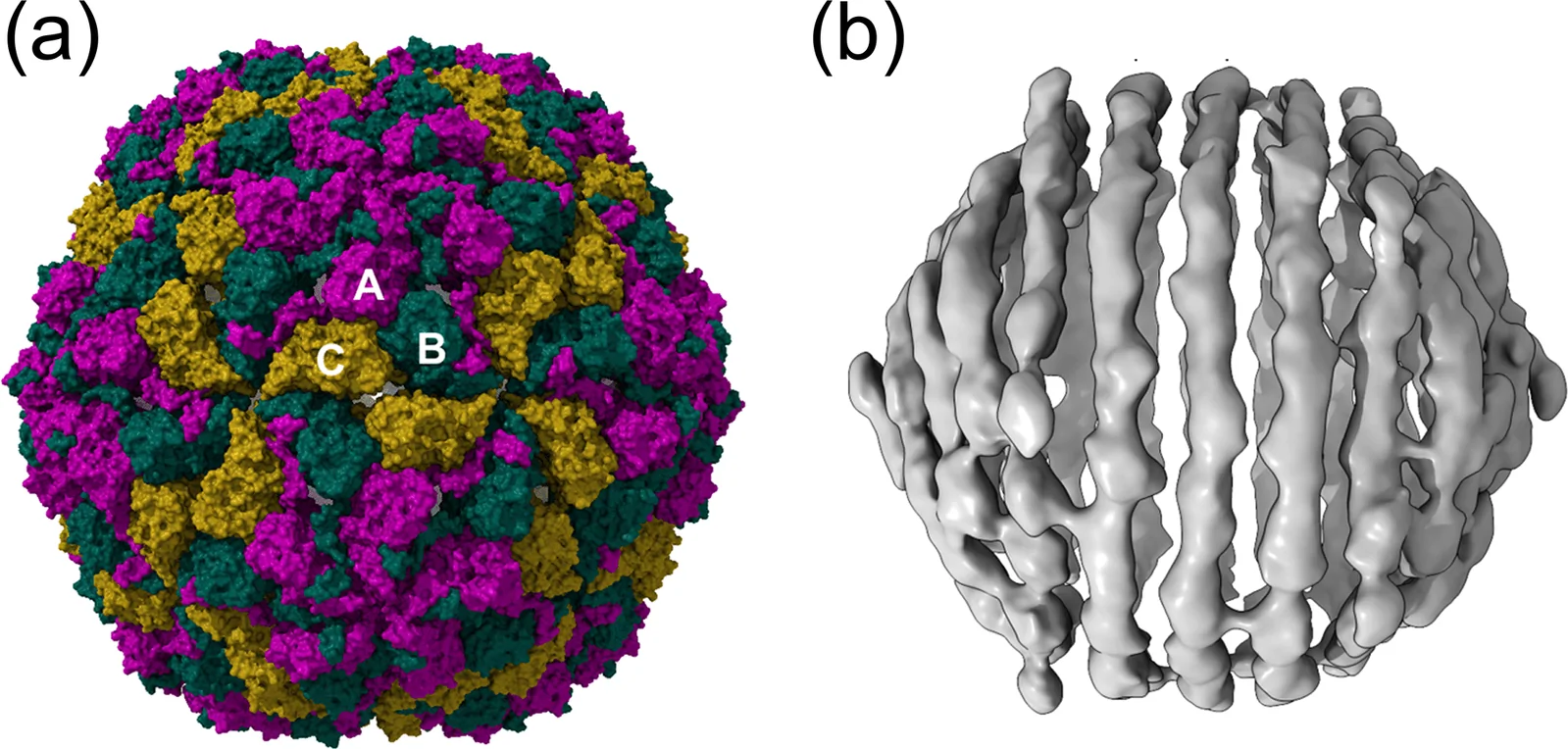
Bacilladnavirid particle. (**a**) Atomic model of the Chaetoceros tenuissimus DNA virus type II capsid. Subunits A, B and C are coloured purple, green and yellow, respectively. (**b**) The outer genome layer. The coil of three turns is visualized in the centre of the image, where the individual parallel turns are separated by ∼28 Å [2].

## Genome

Bacilladnavirids have a circular ssDNA genome of 3.4–6.0 kb with a shorter, complementary linear segment of 0.5–1.0 kb that creates a dsDNA region [4, 5]. However, certain members of the family may lack the complementary segment [6]. The G+C content of bacilladnavirid genomes is 40–50 mol% [3]. Bacilladnavirid genomes encode three to five putative proteins, including the replication-associated protein (Rep) and CP (Fig. 2). For the remaining putative proteins, the functions are unknown. A subset of viruses from the genera *Protobacilladnavirus*, *Diatodnavirus* and *Aberdnavirus* encode a divergent homologue of phospholipase A1, which is predicted to be involved in the lysis of infected diatoms and/or virion entry into the host cells [3]. The Rep of bacilladnavirids contains two domains: the N-terminal HUH superfamily endonuclease and the C-terminal superfamily 3 helicase domain [7], with conserved motifs distinguishing bacilladnavirids from other members of the phylum *Cressdnaviricota* [8]. Bacilladnavirid genomes have a conserved gene order (synteny) with the Rep and CP genes encoded in opposite orientations [3].

**Fig. 2.**
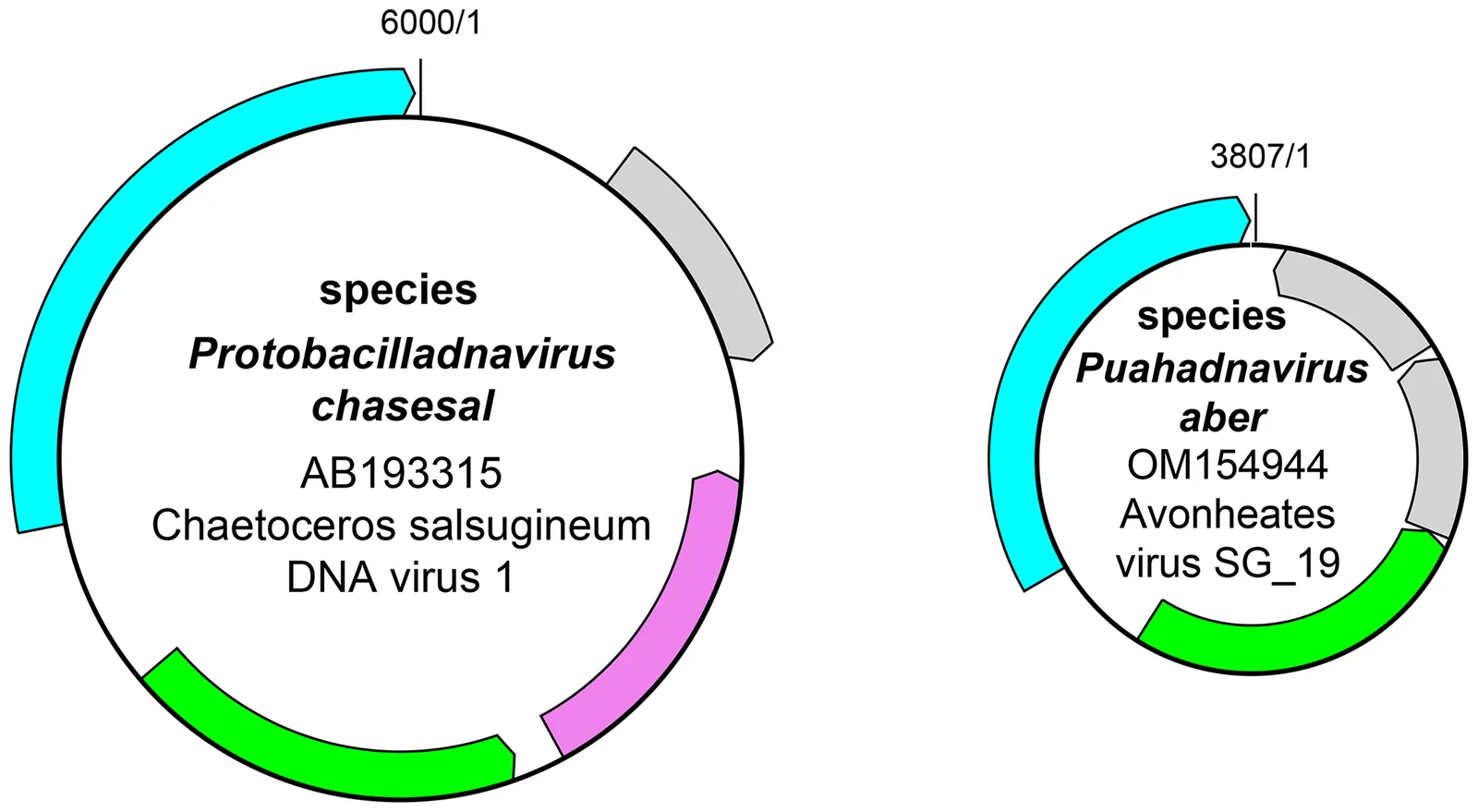
Genome organization of selected bacilladnaviruses. ORF colours: Rep, cyan; CP, green; putative phospholipase, pink; hypothetical proteins, grey.

## Replication

Bacilladnavirid genomes are believed to replicate by the rolling-circle mechanism initiated by the viral Rep and involving one or more host DNA polymerases. Transcriptomic analysis of cells infected with Chaetoceros tenuissimus DNA virus type II shows that several components of the host replisome are significantly upregulated at the later stages of infection (3–24 h post-infection) [9]. Similarly upregulated are host histones, suggesting that chromatinization of viral DNA is important for replication. Bacilladnavirids isolates have a high host specificity [4] and cause lysis of their hosts by an unknown mechanism.

## Pathogenicity

All isolated bacilladnavirids infect diatoms, a cosmopolitan and environmentally important group of unicellular algae (Stramenopiles). Hosts include species from the genera *Chaetoceros* and *Haslea*. Bacilladnavirids have also been detected in environmental samples, such as ocean water and benthic sediments from estuaries, and in samples from aquatic animals; these are most likely due to indirect sampling of diatoms [3]. Viral infection imparts fatal damage to the host in axenic culture but, in nature, diatom populations maintain their sizes despite the presence of bacilladnavirids. This may imply diverse virus resistance strategies but these have not yet been uncovered [10].

## Taxonomy

Current taxonomy: ictv.global/taxonomy. The family *Bacilladnaviridae* includes >6 genera and >21 species.

## Resources

Full ICTV Report on the family *Bacilladnaviridae*: ictv.global/report/bacilladnaviridae.

## Abbreviations

CP: capsid protein
ICTV: International Committee on Taxonomy of Viruses
Rep: replication-associated protein
